# Estimated 24-Hour urinary sodium and potassium excretion in adults in the Northwest Region of Morocco, 2017

**DOI:** 10.1186/s13690-023-01053-y

**Published:** 2023-04-20

**Authors:** Mohamed Idrissi, Naima Saeid, Samir Mounach, Hicham El Berri, Ayoub Al Jawaldah, Fadoua Rahhaoui, Fatima-Zahra Mouzouni, Anass Rami, Kaoutar Benjeddou, Houria Lahmam, Hasnae Benkirane, Mohammed Elmzibri, Khalid El Kari, Abdallah Bagri, Hassan Aguenaou, Latifa Belakhel

**Affiliations:** 1 Ibn Tofaïl University-CNESTEN, Joint Research Unit in Nutrition, Health and Environment, RDC-Nutrition AFRA/IAEA, Rabat-Kénitra, Morocco; 2 Integrative and Computational Neurosciences Team, Laboratory BNRNE, Hassan 1th University, Faculty of Sciences and Technology., BP: 577, Km 3.5 Casablanca Road, Settat, Morocco; 3 Ministry of Health and Social Protection, Division of Non-Communicable Diseases, Rabat, Morocco; 4 Regional Office for the Eastern Mediterranean (EMRO), World Health Organization (WHO), Cairo, 7608 Egypt

**Keywords:** 24-h urinary excretion, Sodium, Potassium, Blood pressure

## Abstract

**Background:**

Excessive sodium (Na) and insufficient potassium (K) intake contribute to a high risk of cardiovascular events. Morocco lacks data on actual Na and K intake in adults. We estimated mean Na and K intake in a Moroccan population of adults residing in the Northwest region using 24-h urinary excretion and examined their association with blood pressure (BP).

**Methods:**

A total of 371 adults from this region, who were recruited for the STEPs Survey Morocco 2017, completed demographic, anthropometric as well as BP data and provided a valid 24-h urine collection according to the standard World Health Organization (WHO) protocol. Multiple Linear Regression analysis was used to examine the association between 24-h urinary sodium (24-hUNa) and 24-h potassium excretion (24-hUK) with BP.

**Results:**

Mean Na excretion was 2794 mg/day and mean K excretion was 1898 mg/day. Overall, only 114 (30.7%) adults met the WHO recommendation for Na intake (< 2000 mg/d) and 31 (8.4%) met the adequate level for K intake (⩾3510 mg/d). There was no association between 24-hUNa and 24-hUK with BP (*P* > 0.05 for all).

**Conclusion:**

Na intake was higher and K intake was lower than WHO recommendations in the study population. There was no association between estimated Na and K intake levels with BP in this population.

## Background

Non-communicable diseases (NCDs) are the leading cause of death in the world. They kill 41 million people each year, accounting for 74% of all deaths globally [1]. Unhealthy diet is a modifiable behavioral risk factor for NCDs and a major contributor to their development [2].

A diet that provides too much Na and too little K can increase BP and ultimately lead to cardiovascular disease (CVD) [3–6]. CVD accounts for most NCD deaths (17.9 million) worldwide each year [1]. In this context, the WHO has recommended a Na intake of less than 2000 mg/day (equivalent to 5 g/day of salt) [7], and a K intake of at least 3510 mg/day for adults [8]. In addition, estimating the ratio of Na to K intake (Na: K) is considered as important as estimating either one separately, and a molar Na: K⩽1 has been suggested to achieve adequate health benefits [7]. In Morocco, 38% of premature deaths are attributed to CVD [9]. This high rate could be explained in part by the high Na and low K levels reported in previous studies using dietary surveys or spot urinary methods [10, 11]. More studies based on 24-h urine collection, which is the recommended method for estimating mean Na intake [12], are needed in the Moroccan population. Such research is important to implement the national salt reduction strategy to prevent and control NCDs in the country [13]. In this regard, and based on WHO guidelines [14], Morocco's Ministry of Health and Social Protection conducted the STEPs Survey Morocco 2017 (SSM 2017) to update national data on NCD risk factors. The first aim of this study in adults in the Northwest region, who participated in the SSM 2017, was to estimate Na and K intake using 24-h urinary excretion. The second aim was to examine the association between estimated Na and k intake with BP level.

## Materials and methods

### Data source

Data are from adults residing in the Northwest region (Rabat-Sale-Kenitra), recruited for the urinary validation study of the national SSM 2017. This survey aimed to collect data on NCD risk factors in all regions of Morocco using the WHO STEPwise guidelines [14]. The fieldwork was conducted from March 1st, 2017 to June 3rd, 2017. The study was approved by the Biomedical Research Ethics Committee of the Faculty of Medicine and Pharmacy of Rabat, Morocco. All subjects signed their consent before participation.

### Recruitment and sampling techniques

Eligible subjects for the SSM 2017 were selected using the WHO STEPs sampling design based on the country's most recent census data, the 2014 Moroccan census [11]. It is a stratified three-stage cluster sampling procedure (cluster, household and individual). The same sampling design was used for this study. First, a list of 33 clusters (a cluster is a geographic area composed of an average of 50 households) was randomly selected from the 609 clusters initially identified to recruit participants for the survey in the Northwest region. Second, 25 households were randomly selected from each cluster using the geographic map of each cluster; from a starting point on the cluster boundary sheet, the interviewer drew households to be surveyed in a clockwise direction, using a grid of two households. Third, one eligible adult was randomly selected from each household using the E-step computer program. A total of 825 adults (594 from urban areas, 231 from rural areas) were screened to determine their willingness and eligibility to provide 24-h urine collection and participate in the study. Individuals on a salt-free diet or with self-reported renal dysfunction and menstruating women were excluded from this study.

### Data collection

All field data were collected by trained staff made up of three nutrition students, three nurses, and one physician. Standard protocols and instruments of the WHO STEPwise Surveillance Approach were used and data were collected under the supervision of Morocco’s Ministry of Health and Social Protection. The E-step application system was used for data entry.

### Demographic and health characteristics

Self-reported data on demographic characteristics, health behaviors, and current use of medications to treat hypertension, diabetes, and hypercholesterolemia were recorded using standardized questionnaires [11]. Subjects were classified into three subgroups based on the number of years spent in school (never been to school, ≤ 9 years, and > 9 years). Information on participants' health behaviors and current medication use was collected based on their "yes" or "no" responses to standard questionnaires [11].

### Anthropometric and blood pressure measurements

Subjects' height (cm) and weight (Kg) were measured according to standard WHO protocols [15]. These two variables were used to estimate body mass index (BMI), calculated as weight in kilograms divided by height in meters squared. Subjects were then classified according to their BMI into (a) underweight (< 18.5 kg/m^2^), (b) normal weight (18.5─24.9 kg/m^2^), (c) overweight (25.0─29.9 kg/m^2^), and (d) obese (≥ 30.0 kg/m^2^) [16]. Three recordings of brachial systolic blood pressure (SBP) and diastolic blood pressure (DBP) were averaged to determine the mean BP values for each participant. BP was measured in participants in the seated position using a calibrated digital sphygmomanometer (Digital Automatic Blood Pressure Monitor, Spengler ES 60) with appropriate cuff sizes. Before BP measurement, participants were allowed to rest for at least 5 min [14]. Hypertension was defined as those with a SBP ≥ 140 mmHg or DBP ≥ 90 mmHg, or those who reported using antihypertensive medications.

### Twenty-four-hour urine collections

Standardized oral and written instructions on 24-h urine collection were presented to each participant after distribution of the kit containing all collection materials [15]. Participants were asked to collect the 24-h urine sample during the weekend, starting the collection on Saturday morning and finishing on Sunday morning. The first morning voiding was discarded, and then urine was collected for the next 24 h, including the first voiding the next morning. Participants recorded the start and end time of the collection period.

The specimens were transported to the laboratory in thermoelectric coolers. Emphasis was placed on the importance of collecting the last void and trying not to lose any drops. Participants were instructed to keep the samples in a cool, dark place away from direct sunlight. Once the collection was returned, the total volume was recorded by the laboratory technicians and four 5 ml aliquots were obtained after shaking. Immediately, the samples were frozen: two aliquots were stored at -21° for Na, K and creatinine analysis, while the other two aliquots were stored at -80° in the laboratories of the "Joint Research Unit in Nutrition and Food-Rabat" as backup. A valid 24-h urine collection was defined as (1) total urine volume 400 ml ≤ V ≤ 3600 ml; (2) loss of no more than one drop; and (3) collection period between 22 and 26 h [17].

### Urinary analysis

Urinary Na and K concentrations were measured by inductively coupled plasma mass spectrometry (ICP-MS; Thermo Scientific XSERIES2), and creatinine concentration by the Jaffe method using the Cobas C311 (Roche diagnostic, Meylan-France). An international reference material (Seronorm TM Trace Elements Urine) was used to control and validate Na and K measurements. Repeated measurements of urinary Na, K, and creatinine showed low analytical imprecision with a coefficient of variation of 1.5%, 2.5% and 1.2%, respectively. The total concentration of Na, K, and creatinine excreted during 24 h was normalized by multiplying the analyte concentration by the total urine volume.

### Statistical analysis

Baseline demographic and health characteristics of participants were examined overall and by gender. The 24-hUNa and 24-hUK were assessed for the entire sample and after classification by age, education level, geographic area, and BMI. Continuous variables were presented as mean (standard deviation (SD)) and median (interquartile range (IQR)). Categorical variables were reported as percentages (95% confidence interval (95% CI)). The Independent Samples *t* Test, Mann–Whitney U test, or One-Way Analysis of Variance (ANOVA) were used to compare means of continuous variables according to the case. ​​For categorical variables, Pearson's χ^2^-test was used. Percentages of the population with urinary Na excretion < 2000 mg/day, K excretion ≥ 3510 mg/day, and Na: K excretion ⩽1 were calculated. All values were estimated using the bootstrap test. Multiple Linear Regression analysis was used to examine the association between 24-hUNa and 24-hUK with BP. In addition, factors known to be associated with BP, including age, sex, and BMI [18–20], were used to adjust the association. Data from the Multiple Linear Regression were presented as a regression coefficient (β) with 95% CI and corresponding *p* values. All statistical tests were two-tailed, a *p* < 0.05 was used for test significance. Statistical Product Service Solutions-SPSS 21 and Excel 2019 for data analysis and presentation were used.

## Results

### Participant flow for the urinary validation study, SSM 2017

**Fig. 1:**
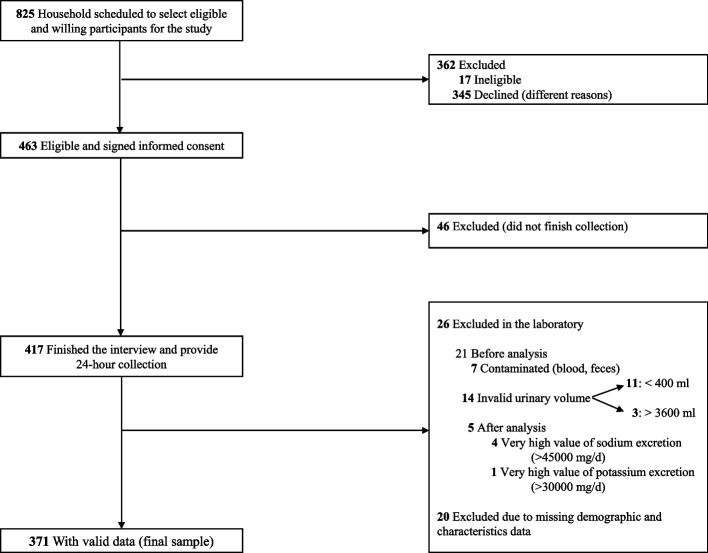
Participant recruitment flow chart, urinary validation study, SSM 2017

Of the 463 adults who agreed to participate, 92 were excluded (46 did not complete the 24-h urine collection, 26 provided invalid samples, and 20 had missing data) (Fig. 1). The remaining 371 adults constituted the final study sample (Fig. 1).

### Characteristics of participants

Of the 371 participants, 189 (50.9%) were aged between 18 and 49 years old, 199 (53,6%) had never attended school, and 258 (69.5%) were from urban areas (Table 1). One hundred and thirteen (30.5%) were obese. Hypertensive subjects represented 31.0% and diabetics 18.0% of the study population (Table 1).

**Table 1:** Characteristics of the study population by gender in adults in the northwest region of Morocco, urinary validation study, SSM 2017

Characteristics	Overall (*n* = 371)	Men (*n* = 132)	Women (*n* = 239)	*P* value
	% (95% CI)	% (95% CI)	% (95% CI)	
Age, years				
18–49	50.9 (46.1–56.1)	44.6 (36.0–53.3)	54.4 (48.2–60.8)	
50–94	49.1 (43.9–53.9)	55.4 (46.7–64.0)	45.6 (39.2–51.8)	0.073^a^
Education attainment				
Never been in school	53.6 (48.3–58.6)	50.0 (41.3–58.8)	55.6 (49.2–61.9)	
≤ 9 y	31.7 (26.9–36.7)	29.4 (21.5–37.6)	32.9 (26.9–39.0)	
> 9 y	14.7 (11.1–18.3)	20.6 (13.8–28.1)	11.5 (7.6–15.8)	0.067
Geographic area				
Urban	69.5 (65.0–74.1)	63.2 (55.3–72.1)	72.6 (66.9–78.0)	
Rural	30.5 (25.9–35.0)	36.2 (27.9–44.7)	27.4 (22.0–33.1)	0.080
BMI				
< 18.5^c^	-	-	-	
18.5–24.9 25.0–29.9	31.8 (25.3–37.4) 37.7 (32.9–42.6)	48.4 (36.7–60.3) 36.2 (28.0–44.5)	22.8 (15.9–30.1) 38.6 (32.5–44.8)	
≥ 30.0	30.5 (25.9–35.0)	15.4 (9.4–21.9)	38.6 (32.5–44.8)	< 0.001
Salt reduction	4.0 (02.2–06.2)	0.8 (0.01–2.5)	5.8 (3.0–8.9)	0.019
Curent cigarette smoking	11.4 (8.3–14.7)	12.7 (7.0–18.8)	10.7 (7.0–14.9)	0.566
Current alcohol use	1.7 (0.3–3.3)	0.9 (0.01–2.7)	2.2 (0.5–4.5)	0.389
Hypertension	31.0 (26.4–35.6)	32.6 (24.6–40.7)	30.1 (24.4–36.2)	0.625
Diabetes	18.1 (13.9–22.6)	10.4 (4.9–16.7)	22.5 (16.8–28.5)	0.010
Drug use				
Antihypertensive	17.8 (13.9–21.9)	10.1 (5.1–15.5)	21.9 (16.8–27.4)	0.005
Anticholesterol	1.1 (0.3–2.2)	0.8 (0.01–2.7)	1.3 (0.01–2.9)	0.689
Antidiabetes	3.9 (2.0–6.2)	1.6 (1.2–0.01)	5.1 (2.5–8.1)	0.105
	Mean (SD)	Mean (SD)	Mean (SD)	
Urinary measurements				
Creatinine (mg/day)	1096 (421)	1264 (465)	1005 (365)	< 0.001^b^
Urine volume (ml/day)	1360 (600)	1280 (580)	1400 (620)	0.045

Values are percentages (95% confidence interval; 95% CI) for categorical variables and means (standard deviation; SD) for continuous variables

All values were calculated using bootstrap test

The comparison between men and women was done using: Pearson's χ^2^ test for categorical variables ^a^ and the Independent Samples t-test for continuous variables ^b^

There were only 4 subjects in the underweight category, so they were excluded from the BMI category analysis because the estimate is statistically unreliable ^c^

*BMI* Body mass index.

### 24-h urinary sodium excretion in adults in the Northwest region of Morocco, urinary validation study, SSM 2017

**Fig. 2:**
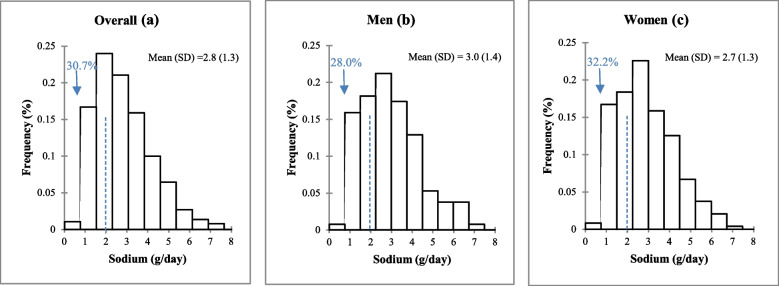
Frequency distribution of 24-h urinary sodium excretion in adults in the Northwest region of Morocco, urinary validation study SSM 2017. (**a**) Overall, (**b**) Men, (**c**) Women. *SD* Standard deviation

Overall, mean Na intake was 2794 mg/day (SD, 1394) (equivalent to 7.1 g/day (3.5)), with men and women having similar values (Fig. 2). Individuals aged 18–49 years consumed significantly more Na than individuals aged 50 years and older (*p* = 0.026) (Table 2). However, there were no significant differences in estimated Na intake between individuals based on geographic area, education level, and BMI categories (Table 2).

**Table 2:** Mean and median 24-h urinary sodium and potassium excretion by gender, urinary validation study, SSM 2017

	Overall (*n* = 371)		Men (*n* = 132)		Women (*n* = 239)	
	Mean (SD)	Median (IQR)	Mean (SD)	Median (IQR)	Mean (SD)	Median (IQR)
Mean sodium excretion						
(mg/day)						
Overall	2794 (1349)	2550 (1780–3650)	2962 (1426)	2665 (1875–3807)	2702 (1301)	2470 (1720–3520)
Creatinine range ¥	2847 (1372)	2615 (1840–3714)	3064 (1410)	2847 (2046–3852)	2738 (1343)	2513 (1732–3578)
Age, y						
18–49	2949 (1351)	2790 (1932–3815)	2956 (1202)	2990 (2080–3740)	2945 (1425)	2700 (1820–4000)
50–94	2633 (1335)	2390 (1695–3325)	2966 (1612)	2530 (1735–3855)	2427 (1089)	2325 (1647–2985)
	0.026^a^	0.007^b^	0.966^a^	0.578^b^	0.002^a^	0.009^b^
Education, y						
Never been in school	2882 (1383)	2696 (1837–3685)	3071 (1451)	2994 (2106–3737)	2790 (1345)	2615 (1740–3654)
9 y	2509 (1273)	2217 (1506–3311)	2544 (1377)	2212 (1423–3579)	2492 (1229)	2222 (1553–3165)
> 9 y	2884 (1394)	2611 (1768–3966)	3116 (1471)	2683 (1907–3958)	2661 (1304)	2417 (1571–3996)
	0.052^c^	0.028^d^	0.159^c^	0.104^d^	0.283^c^	0.230^d^
Geographic area						
Rural	2905 (1538)	2616 (1694–3825)	3054 (1648)	2767 (1688–4092)	2798 (1459)	2587 (1696–3687)
Urban	2735 (1300)	2508 (1763–3558)	2849 (1267)	2571 (1867–3597)	2681 (1306)	2549 (1702–3520)
	0.275^a^	0.556^b^	0.432^a^	0.602^b^	0.548^a^	0.842^b^
BMI						
< 18.5^e^	-	-	-	-	-	-
18.5–24.9	2977 (1566)	2695 (1731–3922)	3036 (1608)	2767 (1780–4015)	2905 (1529)	2632 (1611–3877)
25.0–29.9	2787 (1355)	2599 (1727–3682)	2949 (1422)	2695 (1831–3830)	2706 (1321)	2477 (1678–3483)
≥ 30.0	2742 (1205)	2513 (1980–2573)	2819 (834)	2524 (2205–3360)	2726 (1274)	2513 (1842–3640)
	0.310^c^	0.206^d^	0.415^c^	0.367^d^	0.337^c^	0.214^d^
Mean potassium excretion						
(mg/day)						
Overall	1898 (1046)	1640 (1170–2410)	1966 (1118)	1775 (1092–2525)	1861 (1004)	1610 (1200–2290)
Creatinine range¥	1946 (1065)	1669 (1224–2452)	1995 (1050)	1871 (1336–2441)	1922 (1074)	1610 (1189–2479)
Age, y						
18–49	1931 (1052)	1660 (1190–2470)	1902 (1066)	1640 (1080–2380)	1945 (1048)	1700 (1280–2500)
50–94	1864 (1042)	1610 (1110–2315)	2024 (1168)	1870 (1095–2575)	1766 (948)	1580 (1132–2077)
	0.537^a^	0.457^b^	0.536^a^	0.259^b^	0.167^a^	0.078^b^
Education						
Never been in school	1952 (1069)	1750 (1282–2458)	1892 (981)	1847 (1318–2246)	1981 (1111)	1643 (1263–2572)
9 y	1745 (964)	1517 (1031–2345)	1776 (1153)	1458 (856–2336)	1729 (867)	1544 (1095–2351)
> 9 y	1801 (935)	1542 (1115–2257)	1993 (1041)	1919 (1295–2739)	1617 (796)	1391 (1046–1976)
	0.201^c^	0.201^d^	0.715^c^	0.450^d^	0.094^c^	0.174^d^
Geographic area						
Rural	2072 (1157)	1788 (1366–2498)	1982 (1112)	1841 (1341–2292)	2136 (1192)	1788 (1368–2696)
Urban	1831 (1013)	1598 (1103–2367)	1842 (1024)	1659 (1056–2341)	1825 (1011)	1533 (1115–2381)
	0.044^a^	0.063^b^	0.469^a^	0.559^b^	0.044^a^	0.057^b^
BMI						
< 18.5^e^	-	-	-	-	-	-
18.5–24.9	1933 (1141)	1633 (1067–2269)	1849 (1052)	1649 (1055–2220)	2032 (1242)	1595 (1122–2750)
25.0–29.9	1883 (1094)	1634 (1176–2525)	1942 (1092)	1696 (1214–2597)	1854 (1099)	1477 (1157–2479)
≥ 30.0	1973 (970)	1729 (1318–2498)	2083 (1039)	1906 (1370–2645)	1950 (959)	1696 (1280–2421)
	0.387^c^	0.277^d^	0.465^c^	0.509^d^	0.470^c^	0.342^d^

^¥^Twenty- four-hour creatinine excretion range was 3–25 mmol for women and 6–30 mmol for men [21] (*n* = 355)

The comparison between groups was done using: Independent Samples t Test^a^, Mann–Whitney U test^b^,

One-Way Analysis of Variance (ANOVA)^c^, and Kruskal–Wallis test^d^

^e^There were only 4 subjects in the underweight category, so they were excluded from the BMI category analysis because the estimate is statistically unreliable

*BMI* Body mass index

### 24-h urinary potassium excretion and sodium-to-potassium molar ratio in adults in the Northwest region of Morocco, urinary validation study, SSM 2017

**Fig. 3:**
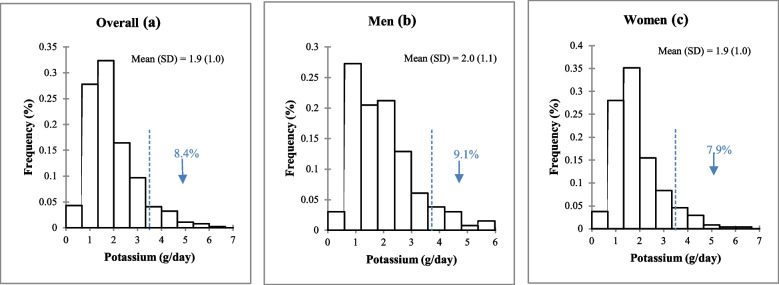
Frequency distribution of 24-h urinary potassium excretion in adults in the Northwest region of Morocco, urinary validation study SSM 2017. (a) Overall, (b) Men, (c) Women. SD; standard deviation

**Fig. 4:**
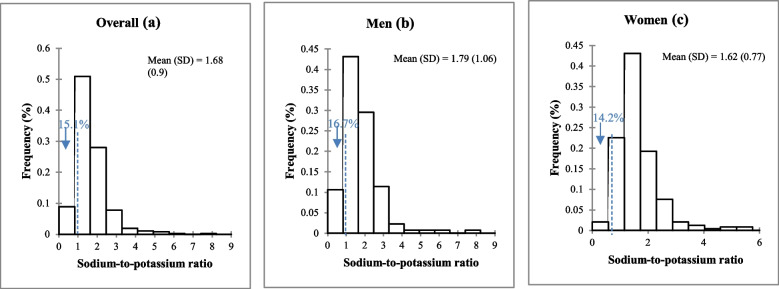
Frequency distribution of urinary sodium-to-potassium ratio excretion in adults in the Northwestern region of Morocco, urinary validation study, SSM 2017. (**a**) Overall, (**b**) Men, (**c**) Women. SD Standard deviation.

Overall, mean K intake was 1898 mg/day (SD, 1046). There were no significant differences by sex (Fig. 3). Similarly, no significant differences were found when categorizing by age, education level, or BMI (Table 2). However, a significant difference was observed between individuals from different geographical areas; subjects from rural areas consumed higher amounts of K compared to subjects from urban areas (*P* = 0.044) (Table 2). The mean Na: K intake in the total population was 1.7 (SD, 0.9). Similarly for Na and K intake, there was no significant difference between genders (Fig. 4).

### Sensitivity analysis

Mean sodium and potassium intake, as well as Na: K, remained unchanged after excluding subjects with creatinine excretion outside the range of 3 to 25 mmol/day for women and 6 to 30 mmol/day for men [21], and after excluding subjects who reported using antihypertensive medication.

### Adherence to recommendations for sodium intake, potassium intake and sodium–potassium ratio in adults in the Northwest region of Morocco, urinary validation study, SSM 2017

**Fig. 5:**
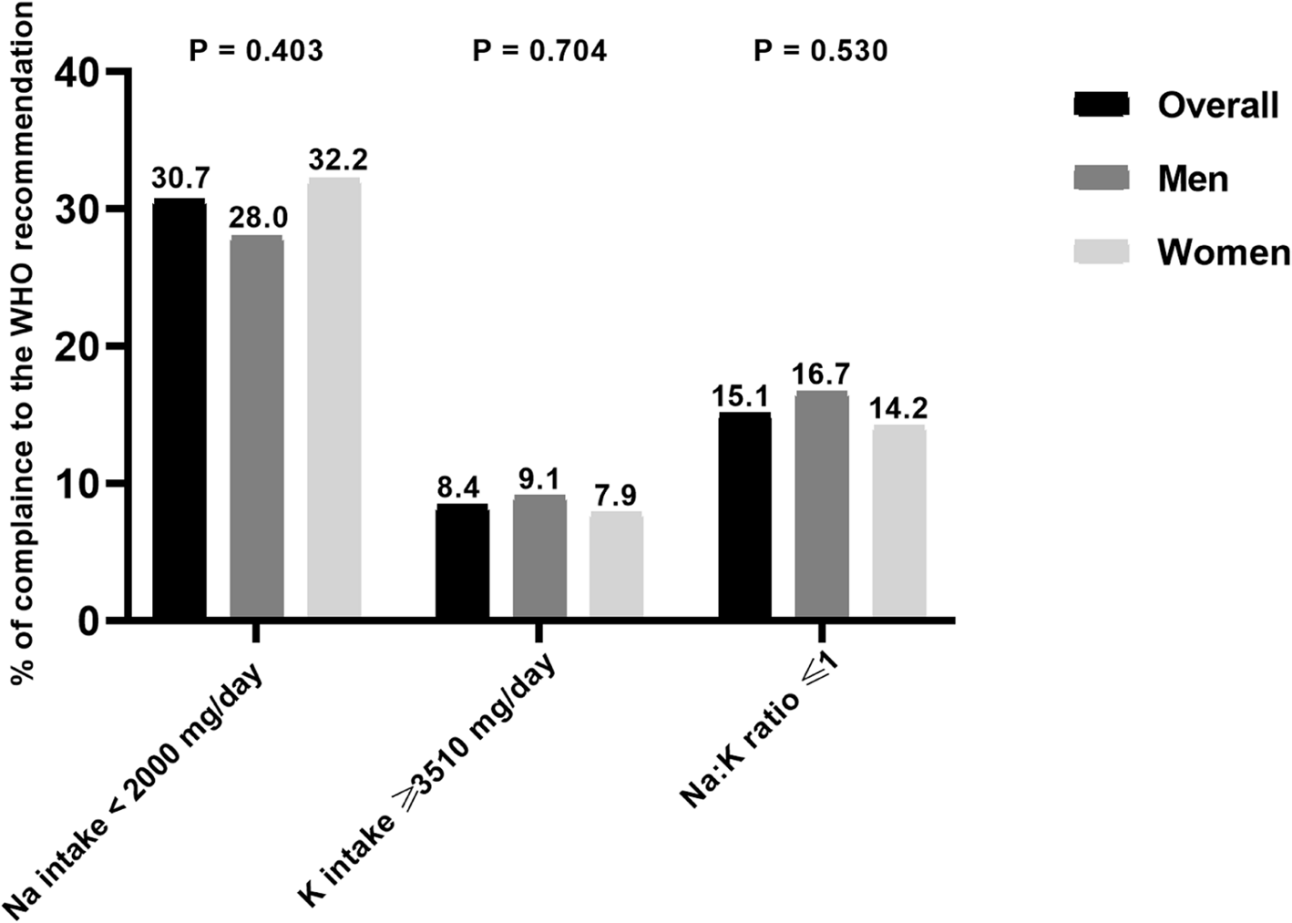
Percentages of adults meeting World Health Organization (WHO) recommended intakes of sodium (< 2000 mg/day), potassium (≥ 3510 mg/day), and sodium-to-potassium ratio (≤ 1), estimated based on 24-h urinary excretion in adults in the Northwestern region of Morocco, urinary validation study, SSM 2017. P values were calculated between genders. Na: K; Sodium-to-potassium intake ratio

Overall, 30.7% of individuals had an estimated Na intake less than the upper limit recommended by the WHO (2000 mg/day), with no significant difference between men and women: 28.0% versus 32.2%, respectively (*p* = 0.403) (Fig. 5). Overall, 8.4% of individuals achieved adequate K intake as recommended by WHO (≥ 3510 mg/day), with statistically similar compliance between men (9.1%) and women (7.9%) (*p* = 0.704) (Fig. 5). Overall, 15.1% of individuals had Na:K⩽1. Again, no significant difference was observed between men and women (*p* = 0.530) (Fig. 5).

### Association of 24-h urinary sodium excretion and potassium excretion with blood pressure

Multiple Linear Regression analysis showed no association between estimated Na and K intake with BP after adjustment for potential confounders (sex, age, and BMI) (*p* > 0.05 for all) (Table 3).

**Table 3:** Multiple Linear Regression analysis to evaluate association of urinary sodium and potassium with systolic and diastolic blood pressure, urinary validation study, SSM 2017

	SBP (mmHg)		DBP (mmHg)	
Variables				
Urinary sodium (mg/day)	-0.001 (-0.03, 0.0003)	*P* = 0.112	-0.001 (-0.002, 0.0004)	*P* = 0.193
Urinary potassium (mg/day)	0.002 (-0.0002, 0.004)	*P* = 0.069	0.001(-0.0001, 0.003)	*P* = 0.072

Values are β coefficient (95% confidence interval) for systolic blood pressure (SBP) and diastolic blood pressure (DBP)

SBP and DBP were used as dependent variables and age, sex, body mass index, urinary sodium, and urinary potassium as independent variables

## Discussion

This is the first study to evaluate 24-hUNa in adults residing in the Northwest region of Morocco. The estimated mean Na intake was within the range reported in countries from the WHO Eastern Mediterranean Region (EMRO). We found no association between Na and K intake with the BP. The few studies conducted in the Moroccan population using methods such as 24-h urine collection, spot urine and dietary surveys showed that estimates of Na intake ranged from 2800 to 4200 mg/day [10, 14, 22]. Our study lines with these findings and adds reliable information on the level of Na intake among Moroccan adults. Our results were also comparable to those reported in the EMRO region. The highest estimates were observed in adults from Iran (4100 mg/day) [23] and Spain (3900 mg/day) [24], while the lowest values were reported in adults from Tunisia [25] and Italy (3200 mg/day) [26], Morocco (2800 mg/day) [22] and the United Arab Emirates (2400 mg/day) [27]. Moreover, less than one third (30.7%) of adults in our study consumed no more than 2000 mg/day of Na and only a few (8.4%) had an adequate K intake of 3510 mg/day. This is consistent with the results of previous studies that reported a lack of commitment to recommended Na and K [22, 22, 24]. This imbalance was also manifested in our study by a high percentage (84.9%) of subjects with Na:K ⩽1, which may reflect changing dietary behavior and food consumption patterns in Morocco. In the last decades, a nutritional transition from the Mediterranean diet to a more "westernized" eating style has been observed in the country [28]. On the one hand, the Moroccan diet is increasingly based on processed foods (high in Na and low in K), especially in urban areas such as the Northwest region [28], with bread being the main source of sodium in the Moroccan diet [29]. On the other hand, the majority of Moroccans (76.3%) consume less than 5 portions of fruits and vegetables per day [11]. This amount of natural food, with a high density of K and other beneficial nutrients, represents a minimum requirement to prevent major diseases such as CVDs and some types of cancers [30]. Overall, these results highlight the relevance of implementing the national strategy to reduce salt consumption in the Moroccan population by 10% by 2029 as a voluntary target adapted by the country to fight against NCDs. In addition, along with the Na reduction strategy, it would be beneficial to take measures to promote K consumption by individuals, by raising awareness for the importance of consuming fruits and vegetables and by making these foods more affordable. Furthermore, no association between urinary Na excretion and BP was found in this study. This lack of association may be explained in part by the narrower range of sodium intakes in our study. For comparison, a positive association between Na intake and BP has been reported in populations with higher sodium intakes than ours (Chinese population (3838 mg/day) [31]; US population (3650 mg/day)[32] vs our population (2794 mg/day)). A main observation made by the authors was that the association with BP was more evident for high levels of Na intake than for low levels [31, 32]. The inconsistency could also be explained by the difference in the methods used to assess Na intake. The authors collected two consecutive 24-h urine samples, whereas we used only one sample. Collecting more than a single 24-h urine sample would take into account intra-individual variations in Na excretion and thus strengthen its association with BP [12]. However, we believe that the relatively low estimate of Na intake would be a determining factor for the lack of association between urinary Na excretion and BP observed in our study.

### Limits

This study has several limitations. Firstly, only one 24-h urine sample was collected, whereas multiple collections are necessary to account for daily intra-personal variations in Na excretion and, therefore, accurately assess habitual Na intake [12]. However, this approach is more difficult for participants because collecting a valid 24-h urine sample can be burdensome [12, 33]. The main objective of this study is to estimate the average Na intake of the population. If provided by an appropriate number of individuals, a single 24-h urine collection would compensate for the variation in intra-personal day-to-day Na excretion and give an accurate estimation of the mean population intake. Secondly, although urine samples were collected over the weekend to facilitate participation in this study, 42% of the initially recruited subjects declined to participate and/or collect the 24-h urine sample. This is likely due to the cultural or psychological burden of the method. The impact of differences in baseline characteristics between the first-recruited population and the final sample on the level of Na and K intake has not been examined, but the final sample remains adequate to estimate Na and K intake in a group of subjects [11, 34]. Finally, the sample size was sufficient for accurate estimation of mean Na and K intake but modest to examine the association with BP levels. Besides the aforementioned limits, our results will help to enrich and fill existing gaps in the national database with accurate information to implement the national salt reduction strategy and promote K intake actions in the country's health programs.

## Conclusion

Na intake was higher and K intake was lower than WHO recommendations in this population of adults in the Northwest region of Morocco. At the estimated levels of Na and K intake in this population, there was no significant association with BP. Longitudinal studies with larger populations to evaluate the association between Na and BP are needed in Morocco. Simultaneously, based on the benefit of sodium reduction on BP, the current data will guide Morocco's Ministry of Health and Social Protection to implement the national strategy to reduce salt intake in the population as a cost-effective intervention to prevent NCDs in the country.

## Data Availability

The data used to support the findings of this study are available from the corresponding author, MI, upon reasonable request.

## Abbreviations

Na: Sodium
K: Potassium
BP: Blood Pressure
WHO: World Health Organization
24-hUNa: Twenty-four-hour urinary sodium excretion
24-hUK: Twenty-four-hour urinary potassium excretion
NCDs: Non-Communicable Diseases
CVDs: Cardiovascular Diseases
SSM 2017: STEPs Survey Morocco 2017
BMI: Body Mass Index
SBP: Systolic Blood Pressure
DBP: Diastolic Blood Pressure
SD: Standard Deviation
IQR: Inter-Quartile Range
95% CI: 95% Confidence Interval
ANOVA: Analysis of Variance
